# What is the yield of malaria reactive case detection in the Greater Mekong Sub-region? A review of published data and meta-analysis

**DOI:** 10.1186/s12936-021-03667-5

**Published:** 2021-03-04

**Authors:** Jacqueline Deen, Mavuto Mukaka, Lorenz von Seidlein

**Affiliations:** 1 Institute of Child Health and Human Development, National Institutes of Health, University of the Philippines, 623 P. Gil St, 1000 Manila, Philippines; 2 Faculty of Tropical Medicine, Mahidol-Oxford Tropical Medicine Research Unit, Mahidol University, Bangkok, Thailand; 3 Centre for Tropical Medicine and Global Health, Nuffield Department of Medicine, University of Oxford, Oxford, UK

**Keywords:** Malaria, Active case detection, Reactive case detection

## Abstract

**Background:**

Reactive malaria case detection involves the screening of those in contact with index cases and is used in countries in the Greater Mekong Sub-region. The yield of reactive case detection, defined here as the percentage of positive malaria cases among potential contacts who were screened, was assessed.

**Methods:**

A literature search was conducted on PubMed to identify studies on reactive case detection in the Greater Mekong Sub-region. Eligible published articles were reviewed and pooled estimates from the studies were calculated, by type of malaria test used.

**Results:**

Eighty-five publications were retrieved, of which 8 (9.4%) eligible articles were included in the analysis. The yield from reactive case detection ranged from 0.1 to 4.2%, with higher rates from PCR testing compared with microscopy and/or rapid diagnostic test. The overall yield from microscopy and/or rapid diagnostic test was 0.56% (95% CI 0.31–0.88%), while that from PCR was 2.35% (95% CI 1.19–3.87%). The two studies comparing different target groups showed higher yield from co-workers/co-travellers, compared with household contacts.

**Conclusion:**

In low malaria transmission settings, the effectiveness of reactive case detection is diminishing. In the Greater Mekong Sub-region, modifying reactive case detection from household contacts to co-workers/co-travellers and from testing to presumptive treatment of targeted contacts, could increase the impact of this approach.

## Background

The countries in the Greater Mekong Sub-region (GMS) maintain health systems for the diagnosis, management, and reporting of malaria cases detected by passive case detection (PCD). Early detection and appropriate treatment of patients who present to health workers is effective in the control of malaria. As malaria cases decline and regions like the GMS advance towards malaria elimination, more intensive methods than PCD are needed to accelerate the interruption of transmission and prevent reversals [1]. Active case detection (ACD) strategies require investigators to visit households, farms and forests and actively look for and treat malaria cases. ACD in the GMS is focused on Migrant Mobile Ethnic and Vulnerable (MMEV) populations, who are at increased risk because of limited access to health-care services [2]. ACD may take the form of reactive case detection (RACD) or proactive case detection (PACD), both of which have been adopted in the GMS countries. RACD involves the screening of those potentially in contact with malaria index cases, including their household members, other community members within a radius around the index case, co-workers and co-travellers. PACD involves the general screening of high-risk populations before they present with malaria.

RACD is labour- and resource-intensive. The diagnoses of the index case needs to be confirmed and the contacts need to be traced, identified, informed, tested and treated, if found infected. Since speedy detection and treatment of infected contacts is of the essence, a ≤ 7-day time window from diagnosis of the index case to the treatment of the contacts is recommended [3]. The yield of RACD, defined here as the number of secondary cases detected per number of contacts investigated, depends on the characteristics of the screened population, including occupation, mobility and the type of contact with the index case. The yield of RACD also varies by how the contacts are selected for screening following the identification of the index case, malaria transmission dynamics and endemicity of the area and the sensitivity of the diagnostic test used. Simulations using data from four villages on the Myanmar–Thailand border showed that RACD using rapid diagnostic tests (RDT) has a limited ability in halting transmission in regions of low and unstable transmission due to high spatial heterogeneity of cases, acquisition of malaria infections outside the village, as well as missing low density, asymptomatic infections, which can make up the majority of infections but cannot be detected with the available diagnostic tools [4]. These simulations may not reflect actual data or be generalizable to other areas of the GMS. To get a better understanding of the current, empirical yield of RACD the available published data on RACD in the GMS were reviewed.

## Methods

PubMed was searched using the following search string: “(malaria) AND (“case detection” OR “1–3-7” OR “screen* and treat*”) AND (“Greater Mekong Subregion” OR Cambodia OR Laos OR Myanmar OR Thailand OR Vietnam OR Viet Nam OR China) AND ((“2010/01/01″[PDat]:”2020/09/15″[PDat]))”, with no language restrictions. Titles and abstracts from the literature search were compiled in Endnote (Thomson Reuters, San Francisco, CA, USA).

Titles and abstracts were initially reviewed. Policy descriptions, commentaries and reviews; studies outside the GMS; qualitative and feasibility studies; and entomologic studies were excluded. The relevant full articles on ACD in the GMS were downloaded and reviewed in detail. Included studies were those wherein the screening for malaria started with a passively identified index case. Furthermore, the article had to report the number of contacts screened and the number who were positive for malaria by microscopy, RDT or PCR. The studies could be prospective or retrospective. Articles that did not start with passively detected index cases or did not report the number of contacts screened and the number who were malaria positive were excluded.

Information from each paper was extracted and entered into an Excel spread sheet (Microsoft® Office 2007, Seattle, WA, USA). The descriptive information included location, study period and characteristics of the index cases and contacts screened. The quantitative data collected included the number positive by microscopy, RDT and PCR.

The primary endpoint of this review is the yield of RACD, defined here as the percentage of positive malaria cases among the contacts who were screened, by type of malaria test performed (microscopy and/or RDT versus PCR). Forest plots of the percentage yields (with 95% confidence intervals) from individual studies were created using Stata 16 (StataCorp, College Station, Texas, USA). The weights contributed by each study were calculated from random effects model directly from the Stata “metaprop” command [5]. I^2^ statistic was used to describe the percentage of variation across studies that is due to heterogeneity rather than chance.

The validity of this systematic review was established by adhering to the pre-defined inclusion and exclusion criteria (described above) to allow comparison across individual studies. We expect differences in field and laboratory methods techniques and included studies that met the basic inclusion and exclusion criteria. Publication bias was not systematically calculated since there is no standard for measuring the expected yield of RACD in the GMS.

## Results

**Fig. 1:**
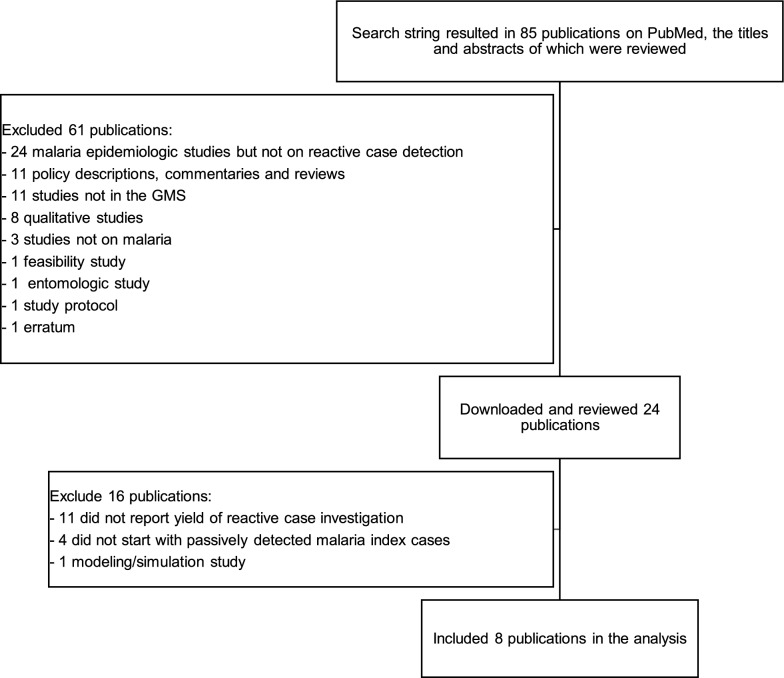
Selection of publications included in the analysis

From a PubMed database search on 15 September 2020, 85 articles were retrieved (Fig. 1). Sixty-one (71.8%) publications were excluded based on the review of the titles and abstracts. Twenty-four articles on ACD in the GMS were identified, downloaded and reviewed in detail. Of the 24, 11 (45.8%) articles that did not report the yield of reactive case investigation, four (16.7%) that did not start with passively detected malaria index cases (these included mass blood surveys and PACD) and one (4.2%) modelling study were excluded. Eight eligible articles [6–13] were included in the analysis, with one reporting on four sub-studies [13]. All eligible articles were in English, except for one (in Chinese) [7], which was translated prior to review.

**Fig. 2:**
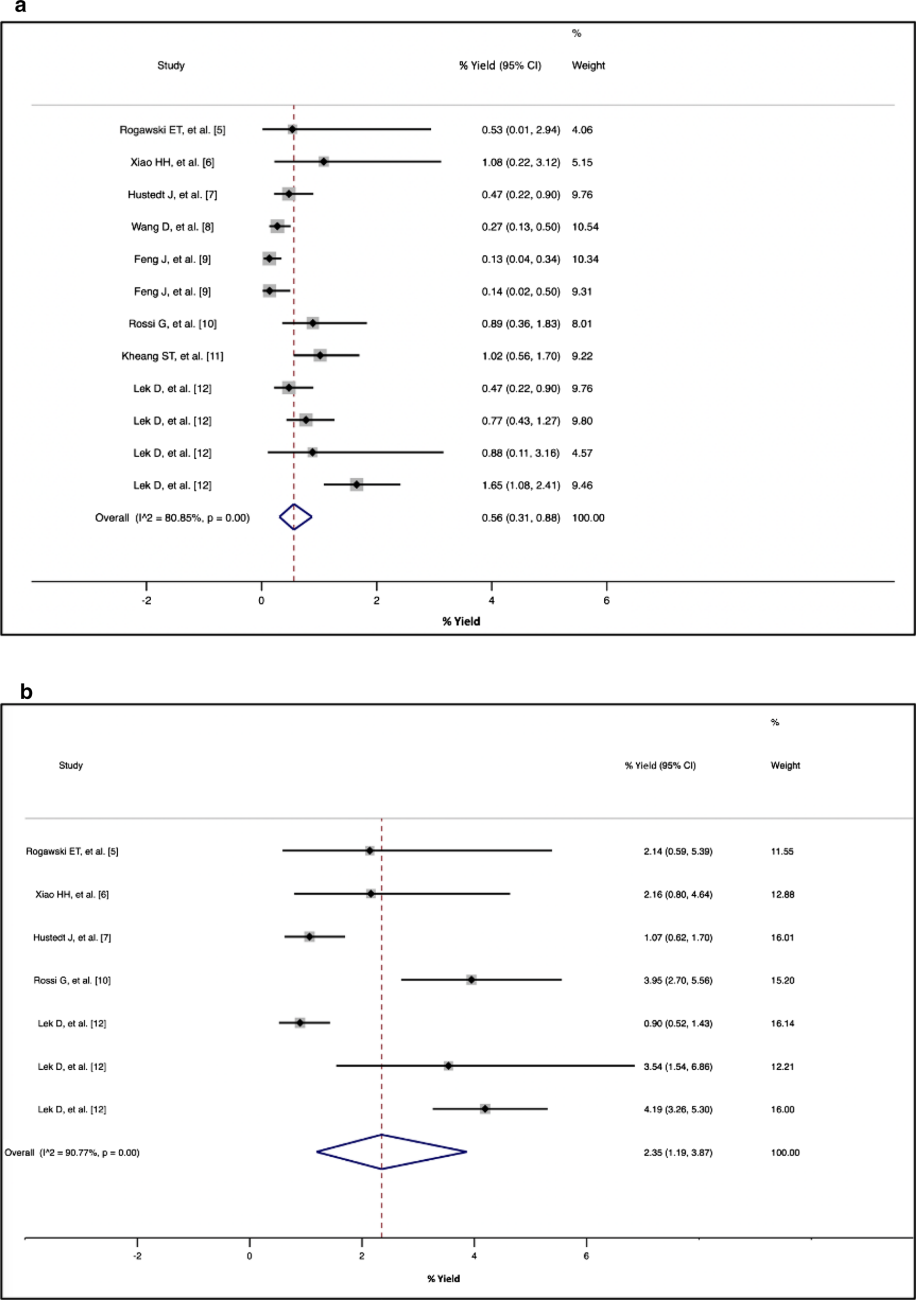
**a**, **b** Metanalysis of reactive case detection (RACD) from published studies in the Greater Mekong Sub-region, by diagnostic test. **a** Percentage yield from RACD using microscopy and/or RDT for screening. **b** Percentage yield from RACD using PCR for screening

The data from the eligible articles were summarized (Table 1). Seven (63.6%) of the 11 study sites were in Cambodia, 3 (27.3%) in China and one (9.1%) in Thailand. The studies were conducted from 2011 to 2018. The number of index cases ranged from one to 639 and the number of contacts screened ranged from 126 to 3,662. The yield from RACD ranged from 0.1 to 4.2%, with higher rates from PCR testing compared with microscopy and/or RDT (Fig. 2a, b). The overall yield from microscopy and/or RDT was 0.56% (95% CI 0.31–0.88%), while that from PCR was 2.35% (95% CI 1.19–3.87%). There was a very high heterogeneity across studies using microscopy and/or RDT and PCR with an I^2^ statistic of 80.9 and 90.8%, respectively. This suggests that 80.9 and 90.8% of the variation across studies is due to heterogeneity. The heterogeneity was statistically significant at *p* < 0.01 for studies using microscopy and/or RDT and PCR. Two studies [11, 12] compared the yield of screening index cases’ household members versus other contacts (co-workers and co-travellers) and found higher rates among the latter (Table 1).

**Table 1:** Summary of studies reporting the yield of malaria reactive case detection

Reference	Location	Study period	Index case(s)	Contacts screened	Yield by		
					Microscopy	RDT	PCR
Rogawski et al. [6]	Bo Rai district, Trat province, Thailand	2011	1 index case, hospitalised with mixed *P. falciparum–P. vivax* infection, identified through passive case detection	126 neighbours within 1 km of the index case were screened 61 soldiers and neighbouring villages with a high proportion of migrants were screened	Microscopist at local malaria clinic—0 detected Expert microscopist—**1 in 187 (0.53%):** *P. falciparum*	Not done or not reported	**4 in 187 (2.14%),** including the *P. falciparum* case detected by microscopy and 3 *P. vivax cases*, which were subsequently confirmed by microscopic examination of more fields
Xiao et al. [7]	Yingjiang County, Yunnan Province (China-Myanmar border), China	2014	Persons living around index cases within a radius of 100 m, 300 m, 500 m, and 1 km were screened	278 persons screened	**3 in 278 (1.08%)** malaria positive	Not done or not reported	**6 in 278 (2.16%)** malaria positive, all within 300 m radius around the index case
Hustedt J, et al. [8]	Pailin, Cambodia	2013–2014	270 index cases (91% *P. vivax*) identified through passive case detection and followed-up at home within 3 days	Household members of index cases were screened. For every 15th index case identified, the five nearest households to the index case’s household were invited to participate For every 30th index case, the ten nearest households were invited to participate	Not done or not reported	**9 in 1898 (0.47%)** members of index and neighbour households: 7 *P. vivax* and 2 *P. falciparum*	**17 in 1596 (1.07%)** members of index and neighbour households: 15 *P. vivax* and 2 *P. falciparum/P. vivax-*mixed
Wang et al. [9]	Four counties in Yunnan Province, (China-Myanmar border), China	2012 to 2014	260 index cases reported and investigated, of which 182/260 (70.0%) had contacts screened	3,662 persons screened	**10/3,662 (0.27%)** malaria positive		Not done or not reported
Feng et al. [10]	China	2013 to 2014	101 cases reported and were categorized as residual non-active foci	2,985 members of index case’s household and neighbouring households within a 300-m radius were screened	Not done or not reported	**4/2,985 (0.13%)** malaria positive	RDT results verified by PCR at the Chinese Center for Disease Control and Prevention
		2015	28 indigenous cases were reported, and active foci response was carried out for all of them	1,447 members of index case’s household and neighbouring households within a 300-m radius were screened	Not done or not reported	**2/1,447 (0.14%)** malaria positive	
Rossi et al. [11]	Chey Saen district, Preah Vihear province, Cambodia	2015 to 2017	194 index cases of *P. falciparum* malaria infection were identified	785 contacts screened: 623 household members of each index case were screened 162 “Co-exposed” individuals, mainly coworkers in settings with a high malaria infection risk, such as forests or plantations, were screened	Not done or not reported	**7/785 (0.89%)** 1/623 (0.2%) household members positive 6/162 (3.7%) co-workers positive	**31/785 (3.95%)** 20/623 (3.2%) household members positive 11/162 (6.8%) co-workers positive
Kheang et al. [12]	Sampov Loun, Cambodia	2015 to 2017	408 index cases identified	1,377 contacts screened, including members of index’s and surrounding household and co-travellers (persons who have been working, traveling, or staying outside of the home village with an index case in the past 3–4 weeks)	**14/1,377 (1.02%)** positive cases (nine *P. falciparum* and five *P. vivax*). All positive cases were identified among index case co-travellers; there were no cases identified among index household members or surrounding household members		Not done or not reported
Lek et al. [13]	Pailin	2013	270 index cases	1898 screened	Not done or not reported	**9 (0.47%)** positive	**17 (0.90%)** positive
	Sampov Loun	2015–2018	639 index cases	1946 screened	Not done or not reported	**15 (0.77%)** positive	Not done
	Preah Vihear	2016–2018	60 index cases	226 screened	Not done or not reported	**2 (0.88%)** positive	**8 (3.54%)** positive
	Oddar Meanchey	2017–2018	192 index cases	1574 screened	Not done or not reported	**26 (1.65%)** positive	**66 (4.19%)** positive

Number of malaria positive in contacts screened (%) by diagnostic test used is indicated in bold

## Discussion

In this analysis of published studies on RACD in the GMS, we found an overall yield from microscopy and/or RDT of roughly one case detected per 200 contacts tested. The yield from PCR was four times higher than microscopy and/or RDT but PCR is generally not used for screening because it is expensive, time- and labour-intensive, and requires advanced laboratory capacity. Critically, the results from PCR testing of samples are not immediately available, delaying the treatment of infected contacts. To achieve the highest impact infected contacts should be diagnosed with an appropriate point of care test and treated immediately.

Standard PCR, as used in the studies reported here, had higher yield than RDT, but has been estimated to miss about half of malaria positives, particularly low density infections [14]. Hopes that “highly sensitive” RDTs could solve these diagnostic challenges turned out to be misplaced. [13] “Highly sensitive” in context of RDTs is rather a misnomer because the additional yield compared to standard RDTs is minimal but the price is roughly twice that of standard RDTs.

Further restricting the yield of the RACD reported here is the primary focus on household contacts, an approach which was found to be effective in China where malaria transmission occurs within the household [3]. This approach may be less appropriate in the GMS where the large majority of infections occurs outdoors, in farms and forested areas. The people at risk are, therefore, those who work in the same location as the index case and not necessarily share the same household [15]. Only two studies in this review compared target groups and found higher yields from screening co-workers and co-travellers than household contacts [11, 12].

This review has several limitations. There were only a few published studies reporting the yield of RACD. Secondly, the studies were done in only some areas of the GMS. The majority of data for this review come from Cambodia with some additional data from China and Thailand. A more complete picture would require the inclusion of more data from Laos and Vietnam. Thirdly, the studies incorporated a wide variety of public health and research methodologies in the tracing and confirmation of contacts, which may question the value of aggregating these different datasets. In the absence of other published sources of information, this is the currently best available method to describe the yield of RACD in the GMS. Assessments of routinely collected data from the GMS malaria control programmes would be important to determine the actual yield of RACD. This review highlights the need for more standardized protocols in RACD, so that results can be compared by location and over time.

The implications for public health are multi-fold. If investigators have to test 200 people to detect a single case, their enthusiasm for RACD is likely to wane quickly. Perhaps it is more promising under the given circumstances to treat contacts presumptively with appropriate schizontocidal anti-malarials, i.e. not including the use of 8-aminoquinolines for radical cure of vivax malaria. Presumptive treatment avoids not only the costs for diagnostic tests which can be more expensive than a course of anti-malarials, but also the risk of missing cases due to the inadequate sensitivity of tests. Such presumptive treatment should probably consist of a full curative regimen using a drug combination to be determined in discussion with each National Malaria Control Program. The disadvantages of presumptive treatment is the reluctance of many contacts to receive treatment in the absence of testing and the increase in anti-malarial consumption leading to an increase in drug pressure. Alternatively, locally-appropriate evidence-based targeting of RACD, such as including household members in villages close to the forest but focusing on occupational contacts who share exposure in time and space with the index case in other areas.

Results from screening of contacts may be needed by national programmes to track progress towards malaria elimination and to support certification of elimination in the longer term. Presumptive treatment of contacts could still be carried-out but with the prior collection of dried blood on filter paper, labelled with the date and location. The dried blood smears could be transported centrally and batched-tested by PCR. The deferred PCR results would be used for identifying areas of continued transmission over time. Collection and PCR testing of dried blood smears will only be feasible in countries with available logistics and funds.

## Conclusion

RACD has been adopted by malaria control programmes in the Greater Mekong Sub-region. Clearly the returns of RACD are diminishing as malaria transmission declines further and malaria elimination comes closer. Malaria control programmes may be faced with the question of whether the limited yield is worth the numerous resources required for RACD. Adapting RACD to presumptive treatment of contacts, with evidence-based targeting could increase the impact of this approach.

## Data Availability

All data generated or analysed during this review are included in this article.

## Abbreviations

PCD: Passive case detection
ACD: Active case detection
MMEV: Migrant Mobile Ethnic and Vulnerable populations
RACD: Reactive case detection
PACD: Proactive case detection
RDT: Rapid diagnostic test
PCR: Polymerase chain reaction
MDA: Mass drug administration
